# Adherence to oxygenation and ventilation targets in mechanically ventilated premature and sick newborns: a retrospective study

**DOI:** 10.1186/1471-2431-13-126

**Published:** 2013-08-19

**Authors:** Marianne Trygg Solberg, Ida Torunn Bjørk, Thor Willy R Hansen

**Affiliations:** 1 Lovisenberg Deaconal University College, Oslo, Norway; 2 Department of Nursing Science, Faculty of Medicine, University of Oslo, Nedre Ullevål 9, Stjerneblokka 0850 Oslo, Norway; 3 Department of Neonatal Intensive Care, Women’s and Children’s Division, Oslo University Hospital, Oslo, Norway; 4 Institute for Clinical Medicine, Faculty of Medicine, University of Oslo, Oslo, Norway

**Keywords:** Newborn infant, Premature infant, Mechanical ventilation, Oxygenation

## Abstract

**Background:**

Ventilator treatment exposes newborns to both hyperoxemia and hyperventilation. It is not known how common hyperoxemia and hyperventilation are in neonatal intensive care units in Norway. The purpose of this study was to assess the quality of current care by studying deviations from the target range of charted oxygenation and ventilation parameters in newborns receiving mechanical ventilation.

**Methods:**

Single centre, retrospective chart review that focused on oxygen and ventilator treatment practices.

**Results:**

The bedside intensive care charts of 138 newborns reflected 4978 hours of ventilator time. Arterial blood gases were charted in 1170 samples. In oxygen-supplemented newborns, high arterial pressure of oxygen (PaO_2_) values were observed in 87/609 (14%) samples. In extremely premature newborns only 5% of the recorded PaO_2_ values were high. Low arterial pressure of CO_2_ (PaCO_2_) values were recorded in 187/1170 (16%) samples, and 64 (34%) of these were < 4 kPa. Half of all low values were measured in extremely premature newborns. Tidal volumes above the target range were noted in 22% of premature and 20% of full-term newborns.

**Conclusions:**

There was a low prevalence of high PaO_2_ values in premature newborns, which increased significantly with gestational age (GA). The prevalence of low PaCO_2_ values was highest among extremely premature newborns and decreased with increasing GA. Further studies are needed to identify whether adherence to oxygenation and ventilation targets can be improved by clearer communication and allocation of responsibilities between nurses and physicians.

## Background

Clinical practice with respect to ventilator management, administration of oxygen, and assessment of oxygenation differs greatly among neonatal intensive care units (NICUs) [1,2]. Ventilator treatment exposes newborns to both hyperoxemia and hyperventilation. The goal of ventilator treatment is to balance gas exchange while minimising trauma to the lung tissue [3]. Adjusting oxygenation and ventilator therapy is challenging, and improved strategies are needed to minimise hyperoxemia [4] and hyperventilation with hypocarbia [5,6] in preterm and full-term newborns. Appropriate oxygenation is achieved by titrating the fraction of inspired oxygen (FiO_2_) and the mean airway pressure (MAP). The aim of appropriate ventilation is to maintain an arterial pressure of carbon dioxide (PaCO_2_) of ~5.3 kPa (40 mm Hg) [7].

Nurses need a target range for oxygen saturation (SpO_2_) in order to titrate FiO_2_ appropriately. SpO_2_ in the 85–95% range excludes hyperoxia [8], but there is no consensus for the optimal saturation target ranges in premature newborns [9]. A collaborative prospective meta-analysis of five ongoing trials in the USA, Australia, United Kingdom, New Zealand and Canada (NeOProM), aimed to establish the optimal SpO_2_ target ranges for extremely preterm newborns [9-11]. Interim results recommend SpO_2_ levels > 90% to avoid mortality [9]. Previous recommendations suggested SpO_2_ levels < 85% [12,13]. There is no evidence or consensus to guide the administration of oxygen in full-term newborns [14].

The Neovent study group found that time-cycled, pressure-limited ventilation was the most common mode currently used for neonatal ventilation. The tidal volume (TV) was usually targeted to 4–7 ml/kg [15]. The same study group found that hypocarbia was relatively uncommon during neonatal ventilation, and they speculated that hypercarbia was more common because of the practice of permissive hypercarbia [15].

Few studies relate arterial oxygen tension values to SpO_2_ targets [8]. It is not known how often the problem of hyperoxemia and hyperventilation occurs in NICUs in Norway. The present retrospective study is the first part of a larger study that aims to discover areas for quality improvement regarding oxygen and ventilator treatment of preterm and sick newborns. The purpose of this study was to investigate the documentation of oxygenation and ventilation among newborns receiving mechanical ventilation in a Norwegian NICU, to report on the following: (1) use of oxygen during ventilator treatment; (2) extent of charted deviations from oxygenation and ventilation targets; and (3) data associated with variations in MAP.

## Methods

### Patients and study design

The setting of this study was a level 4 NICU at Rikshospitalet, Oslo University Hospital, Norway. We retrospectively studied the documentation of oxygen and ventilator treatment practices between July 2010 and November 2011using intensive care charts. Patients who satisfied the inclusion criteria were identified through the NICU proprietary quality control database. Infants were eligible for inclusion if they had been mechanically ventilated for a minimum of 3 hours, and we chose to limit data collection to a maximum of 48 hours for each patient. The sample was grouped by gestational age (GA) into extremely premature (23–28 weeks GA), moderately premature (29–37 weeks GA), full-term (38–41 weeks GA), and newborns > 41 weeks post-conceptual age (defined as GA plus chronological age [10]). The principal diagnoses were categorised using ICD 10 (KITH–Health Affairs) and were: immature lungs, other lung/respiratory problems, circulatory problems, and infection.

Relevant variables for oxygenation and ventilator treatment were defined according to the literature, clinical practice, and discussion with experts in the field. Variables collected and reported in this study were GA, sex, birthweight, diagnoses (infection, lung problems, immature lungs and circulatory problems), peak inspiratory pressure (PIP), positive end-expiratory pressure (PEEP), inspiratory time (TI), FiO_2_, TV, MAP, highest preductal SpO_2_ value, arterial pressure of oxygen (PaO_2_), and PaCO_2._ Data were collected on expiratory TV measured from the ventilator, because this measures the effectiveness of mechanical ventilation [16]. Blood gas analysis was carried out on arterial samples only, because capillary blood gases were deemed to have insufficient reliability for our purposes. This reliability problem relates to newborn infants crying as a reaction to vasopuncture, which frequently presents as rapid changes in PaO_2_ and PaCO_2_[17]. Capillary blood gases were therefore analysed solely for comparison with arterial gases.

The limit of acceptable PaCO_2_ was set at 4.7–5.9 kPa [18]. Normal limits for PaO_2_ were 6–10 kPa for premature newborns [19] and 8–10.7 kPa for full-term infants [18]. Appropriate limits for SpO_2_ for newborns receiving supplemental oxygen were set at 88–93% in premature infants and not above 95% in full-term newborns, according to existing practice guidelines in our unit. The normal limits of TV were considered to be 4–6 ml/kg for premature and 5–8 ml/kg for full-term newborns [20].

### Statistical analyses

Statistical analyses were performed using SPSS version 19.0 (SPSS Inc., Chicago, IL, USA). A research assistant checked data for accuracy. A power analysis resulted in the inclusion of charts from 138 premature and full term newborns. This was based on the assumption that the proportion of newborns with SpO_2_ outside the recommended limit, was approximately 10%, estimated with an accuracy of + / – 5% and calculated with a 95% confidence interval (CI).

Descriptive statistics and frequencies were calculated for GA, weight, number of newborns with measurements of arterial blood gases, 1-hour periods with and without arterial and capillary blood gas measurements (based on 24 1-hour periods per day), PaO_2_, PaCO_2_, and TV. The recorded values for TV were summarised with respect to the two limits that were applicable to premature and full-term newborns in the NICU. Preductal SpO_2_ values were analysed for the trend in mean values over time. The cutoff point for defining hypocarbia was PaCO_2_ < 4.7 kPa, and extreme hypocarbia was defined as PaCO_2_ < 4.0 kPa. When analysing the distribution of arterial blood gases, and comparing the prevalence of high versus low PaO_2_ and PaCO_2_ between the GA groups, we controlled for possible interdependence due to repeated measurements in each individual by using a generalised linear model and Wald’s analysis [21]. We used partial correlation to analyse the correlation between PaCO_2_ and TV [22]. A mixed linear model with repeated measures was used to analyse variations in MAP. Any p value < 0.05 was considered significant.

### Ethical approval

Approval by the Regional Committee for Medical Research Ethics in Norway was not required for this study because data collection was anonymous. Permission for the study was obtained from the Data Protection Officer at Oslo University Hospital and from the director of the NICU.

## Results

### Sample description

The documentation included 4978 hours of ventilator time for 138 newborns. Ventilator support consisted of 4702 hours of conventional mechanical ventilation and 276 hours of oscillation. The minimum duration of ventilator treatment was 5 hours and our predetermined maximum period of study was 48 hours. There were 85 male (62%) and 53 female (38%) infants. GA ranged from 23 to 52 weeks and weight at the time of study entry was 426–5345 g. There were 42 (30%) extremely premature, 42 (30%) moderately premature, 34 (25%) full-term and 20 (15%) newborns aged > 41 weeks. The leading diagnoses were lung immaturity [57 (41%)], other lung problems [43 (31%)], circulatory problems (including congenital heart disease) [32 (23%)] and infection [6 (5%)].

### Use of oxygen during ventilator treatment

**Figure 1:**
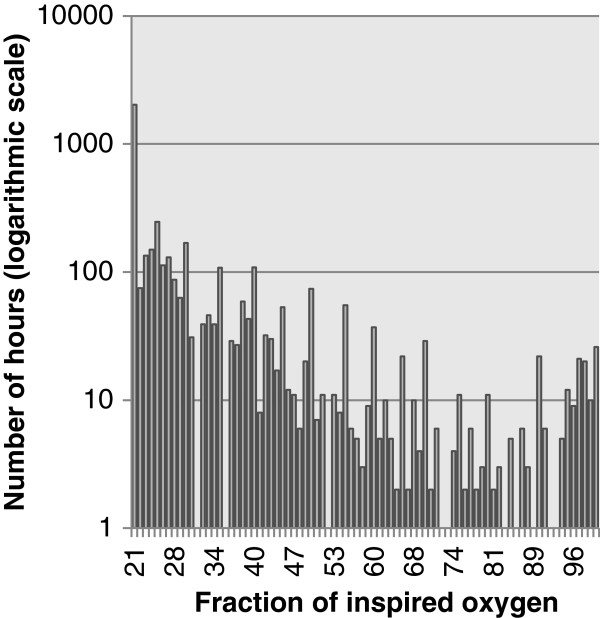
**Distribution of FiO**_**2**_**. ***n* = 4428 hours (138 patients).

Figure 1 summarises the distribution of oxygen concentrations received by the newborns. In total, newborns received 2020 hours of ventilator support with FiO_2_ = 21%. Oxygen was given at a median FiO_2_ of 24% in premature newborns and a median of 21% in full-term newborns. The mean values were respectively 30% (CI 95% = 29.4 - 30.6) and 32% (CI 95% = 31.1 – 32.9).

### Charting of deviations from oxygenation and ventilation targets

Blood samples were taken for arterial blood gas analysis from once an hour, to every 3 to 4 h. Table 1 shows the number of newborns with measurements of arterial blood gases.

**Table 1:** Number of newborns with measurements of arterial blood gases

	**GA 23 - 28**	**GA 29 - 37**	**GA 38 - 41**	**GA > 41**	**Total count**
Total number of newborns with recorded arterial blood gases	41	35	29	16	121
Total number of newborns with recorded arterial blood gases who received FiO_2_ >21%	33	27	19	14	93
Number of newborns with at least one low PaO_2_ (Prem. < 6, full term < 8 kPa)	23	15	15	9	62
Number of newborns with at least one high PaO_2_ (Prem. > 10, full term >10.7 kPa)	8	9	7	9	33
Number of newborns with at least one PaCO_2_ < 4.7 kPa	26	18	18	8	70
Number of newborns with at least one PaCO_2_ < 4 kPa	15	10	7	3	35

Prem. = premature.

### Monitoring and assessment of oxygenation

Table 2 shows the arterial blood gas measurements in 1-hour periods obtained during ventilator treatment.

**Table 2:** Arterial blood gas measurements in 1-hour periods

**Hours of observation**	**GA 23 − 28**	**GA 29 − 37**	**GA 38 − 41**	**GA > 41**	**Total hours observation**
	**n (%)**	**n (%)**	**n (%)**	**n (%)**	**n (%)**
Total hours of observation	1624 (100)	1480 (100)	1112 (100)	762 (100)	4978 (100)
1-hour blocks with listed arterial blood gases	442 (27)	297 (20)	262 (24)	169 (22)	1170 (24)
1-hour blocks without listed arterial blood gases	182 (73)	183 (80)	850 (76)	593 (78)	3808 (76)

Obtained in 121 patients.

Capillary blood gas measurements were obtained in 47 patients. There were 226/4978 (5%) 1-hour periods with recorded capillary blood gases: with 39 (17%) in extremely premature, 94 (42%) in moderately premature, 56 (25%) in full-term, and 37 (16%) in newborns > 41 weeks old. Analysis comparing the frequency of capillary and arterial blood gas sampling showed that arterial blood gas sampling was most common. Wald’s analysis indicated significant differences between the GA groups in the frequency of using arterial blood gases (p = 0.02). Further analysis showed that samples for arterial blood gas analysis were withdrawn significantly more often from the extremely premature versus moderately premature infants (p = 0.004).

Table 3 shows the distribution of low, normal and high PaO_2_ values according to GA in newborns who received oxygen supplementation.

**Table 3:** **PaO**_**2 **_**values in 1-hour periods when FiO**_**2 **_**was >21%**

	**GA 23 − 28**	**GA 29 − 37**	**GA 38 − 41**	**GA > 41**	**Total count**
	**n (%)**	**n (%)**	**n (%)**	**n (%)**	**N (%)**
No. of low PaO_2_ values (Prem. < 6, full term < 8 kPa)	57 (24)	50 (31)	71 (60)	30 (33)	208 (34)^a^
No. of normal PaO_2_ values (Prem. 6–10, full term 8–10.7 kPa)	170 (71)	91 (57)	24 (20)	29 (32)	314 (52)
No. of high PaO_2_ values (Prem. > 10, full term >10.7 kPa)	12 (5)	19 (12)	24 (20)	32 (35)	87 (14)^b^
Total count	239 (100)	160 (100)	119 (100)	91 (100)	609 (100)

Obtained in 93 patients.

^a^Data from newborns with circulatory problems were included in the count of low PaO_2_ values.

^b^Of the high PaO_2_ values, 28 (32%) were >14.6 kPa.

Although extremely premature infants had relatively few high PaO_2_ values, the percentage of high value samples increased with GA. There was a significant difference between GA groups with regard to the risk of low versus normal PaO_2_ values (p < 0.001) and normal versus high values (p < 0.001). Further calculations showed that the odds ratio (OR) of normal PaO_2_ (compared to high PaO_2_) in newborns of GA 23–28 weeks was 16 (95% CI = 6.5–40) times greater than that in newborns of GA > 41 weeks.

**Figure 2:**
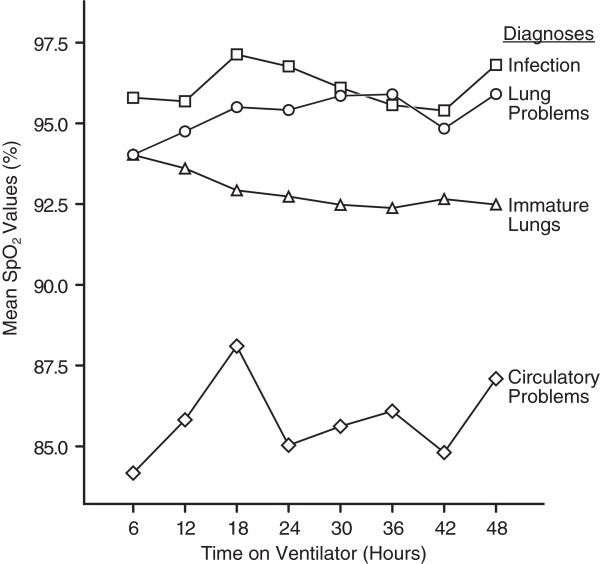
**Mean SpO**_**2 **_**trends by diagnosis.**

The mean SpO_2_ trends over time are shown in Figure 2.

### Monitoring and assessment of ventilation

Low PaCO_2_ values were recorded in 187/1170 (16%) samples (70 patients). When the cutoff for low PaCO_2_ was set at 4 – 4.7 kPa, there were 42 (34%) low PaCO_2_ values in extremely premature, 30 (24%) in moderately premature, and 35 (28%) in full-term newborns, and 16 (13%) in newborns aged > 41 weeks. The analysis showed no significant difference in the occurrence of low PaCO_2_ between GA groups (p = 0.639). However, when the cutoff value was reduced to < 4 kPa (*n* = 64), there was a significant difference (p = 0.015) between the groups. The OR of PaCO_2_ < 4 kPa in newborns of GA 23–28 weeks was 5 (95% CI = 1.6–15) times greater than that in newborns of GA > 41 weeks.

For premature infants of GA 23–37 weeks, there were 2374 recorded TV measurements in 1-hour periods, with a median TV of 4.7 ml/kg. Less than half of the measurements (*n* = 1058) were within the normal reference range of 4–6 ml/kg, and 520 (22%) were > 6 ml/kg. There was no significant correlation between PaCO_2_ and TV (*r* = 0.007, p = 0.87). Full-term newborns had a total of 1535 TV measurements in 1-hour periods, with a median TV of 6.1 ml/kg. More than half of all measurements (*n* = 883) were in the normal range, while 301 (20%) were > 8 ml/kg. There was a significant weak negative correlation between PaCO_2_ and TV (*r* = −0.12, p = 0.03).

### Data associated with variations in MAP

A linear mixed effects model was used to estimate variations in MAP using the independent variables PEEP, TI and PIP (Table 4).

**Table 4:** Mixed-model repeated-measures analyses for variations in MAP

	**Estimate**	**95% confidence interval**		**Sig (p)**
		**Lower**	**Upper**	
Change over time	6.7	1.87	11.54	0.007
PEEP	- 4.43	- 11.76	2.91	0.237
Hours×PEEP	0.12	- 0.26	0.50	0.546
TI	27.77	12.01	43.52	0.001
Hours×TI	- 0.67	- 1.27	- 0.08	0.026
PIP	23.81	17.68	29.94	0.000
Hours×PIP	- 0.55	- 0.79	- 0.31	0.000

Dependent variable: MAP.

Table 4 shows that there was a significant correlation between MAP variability, TI and PIP, which decreased with the passage of time. There was no significant correlation between MAP variability and PEEP. This suggests that changing PEEP was not used as a strategy to adjust MAP and thereby affect oxygenation. The two groups of premature infants received PEEP with a mean pressure of 4.7 cm H_2_O (CI 95% = 4.67 – 4.73), and full-term newborns received PEEP with a mean pressure of 4.9 cm H_2_O (CI 95% = 4.85 – 4.95).

## Discussion

The purpose of oxygen administration in the NICU is to prevent free radical damage [23]. In the present study, the median FiO_2_ in premature newborns was lower (24%) than in the study by van Kaam et al., who found a median FiO_2_ value of 28% during conventional mechanical ventilation [24]. Furthermore, we found that newborns achieved acceptable mean SpO_2_ during the observation period (Figure 2). Targeting saturation to 88–93% in premature newborns and not more than 95% in full-term newborns has been a goal for several years [25,26]. However, these targets have not yet been fully agreed upon, because the results of ongoing randomised trials testing high versus low SpO_2_ targets are still pending [9,10,27]. Our calculation of mean SpO_2_ may have masked episodes of hypoxemia or hyperoxia when nurses titrated FiO_2_. Nevertheless, it suggests that the goal for saturation targets was met to a large extent. Using SpO_2_ alone to guide decisions concerning oxygen administration is not evidence-based practice. Thus, nurses also have to assess skin colour, heart rate, and values from blood gases as well transcutaneous O_2_ measurements [28].

Although it has been suggested that entrenched clinical practices and cultures make it difficult to change the use of oxygen [29], our results showed that extremely premature newborns did not receive excessive amounts of oxygen (Tables 1 and 3). The incidence of hyperoxemia increased with GA. Because of the haemoglobin oxygen dissociation curve [12], sick newborns with pulmonary hypertension are at higher risk of developing high levels of hyperoxemia when they are treated with an oxygen saturation of ~95%. It is common practice in NICUs that nurses wait until an infant is stable before withdrawing arterial blood for gas analysis, and this practice will affect the results. In addition nurses’ workload is an important factor in the achievement of SpO_2_ goals and appropriate oxygen management in the NICU. There is evidence that compliance with saturation targets is improved with higher nurse: patient ratios [30]. The good results for oxygen management in the NICU in the present study may in part have been due to the practice of having a 1:1 nurse:patient ratio for all infants on mechanical ventilation.

Our study revealed that low PaCO_2_ values occurred most commonly in extremely premature infants. Hypocarbia may cause cerebral vasoconstriction, resulting in decreased oxygen delivery to the brain [7,10]. Moreover, in extremely preterm infants, PaCO_2_ in the normal range seems to yield the best electroencephalography activity [31]. PaCO_2_ can be regulated by controlling the minute ventilation with TV, or the ventilator rate [3]. It is therefore noteworthy that we found high TVs in 22% of preterm infants and 23% of newborns aged >37 weeks. Nevertheless, the median TV of 4.7 ml was lower than the 5.3 ml recorded in the study of van Kaam et al. [24]. Mechanical ventilation using high TVs is known to cause lung damage [32]. We did not find any significant correlation between PaCO_2_ and TV in the premature infants, and only a weak negative correlation in full-term infants. This suggests that the relationship between lung physiology and what happens during respirator treatment may not be simple.

Our results showed that variations in PEEP had no significant effect on MAP variability (Table 4). Changing PEEP is often an effective way to adjust oxygenation [33] and to regulate MAP for experienced clinicians who can accurately assess changes in measured data and calculate the impact of any adjustments [34]. The premature newborns in our study had a PEEP mean value of 4.7 cm H_2_O which was similar to that observed in the study of van Kaam et al., although they suggested that PEEP values >7 cm H_2_O might be protective for the lungs [24].

It is suggested that maintaining appropriate oxygenation is hindered by insufficient communication of unit policies as well as personal bias about the best practice [35]. Control of oxygenation and ventilation is crucial during mechanical ventilation. Therefore, further studies should identify how communication and allocation of responsibilities between nurses and physicians can reduce the incidence of hypocarbia and hyperoxemia.

One limitation of this study was that we had no record of fluctuations in oxygenation levels, nor observations of how soon after blood gas analyses adjustments were made. Regarding the analysis of the mean SaO_2_, it would have been helpful to include an analysis that indicated the uncertainty in the results. However, because of the volume of repeated measurements for each individual, a standard box plot could not be used. Another weakness of the study was related to the high TV values, which were presented as a general occurrence regardless of how many high values there were from each individual patient.

## Conclusions

We showed that, in general, premature newborns were treated within the desired limits of SpO_2_, and few high PaO_2_ values were noted. The occurrence of high PaO_2_ values increased significantly with GA. Many recorded TVs were too high and hypocarbia during ventilation was more common in the extremely premature infants.

## Abbreviations

FiO2: Fraction of inspired oxygen; GA: Gestational age; MAP: Mean airway pressure; NICU: Neonatal intensive care unit; OR: Odds ratio; PEEP: Positive end-expiratory pressure; PIP: Peak inspiratory pressure; TI: Inspiratory time; TV: Tidal volume.

## Competing interests

The authors declare that they have no competing interests.

## Authors’ contributions

MTS formulated the hypotheses and designed and conducted the research, analyzed the data, and wrote the paper. ITB participated in the formulation of the hypotheses and study design, co-authored the paper. TWRH participated in the formulation of the hypotheses and study design, offered advice on data collection, and co-authored the paper. All authors read and approved the final manuscript.

## Authors’ information

MTS, RN, Intensive Care Nurse Specialist, Master of Nursing Science, PhD student, Faculty of Medicine, University of Oslo, Norway.

ITB, RN, Master of Nursing Science, PhD. Professor Institute of Nursing and Health Sciences, Faculty of Medicine, University of Oslo, Norway. ITB is a supervisor of MTS.

TWRH, MD, PhD, Professor, Department of Neonatology, Women’s & Children’s Division, Oslo University Hospital – Rikshospitalet, Norway and Institute for Clinical Medicine, Faculty of Medicine, University of Oslo, Norway. TWRH has been the senior supervisor of MTS.

## Pre-publication history

The pre-publication history for this paper can be accessed here:

http://www.biomedcentral.com/1471-2431/13/126/prepub
